# First person – Julie Frion

**DOI:** 10.1242/bio.060144

**Published:** 2023-09-21

## Abstract

First Person is a series of interviews with the first authors of a selection of papers published in Biology Open, helping researchers promote themselves alongside their papers. Julie Frion is first author on ‘ CRISPR/Cas9-mediated knockout of the ubiquitin variant UbKEKS reveals a role in regulating nucleolar structures and composition’, published in BiO. Julie is a PhD Student in the lab of Professor François-Michel Boisvert at PRAC-Université de Sherbrooke, Canada, studying a new protein encoded by what was previously thought to be a pseudogene.


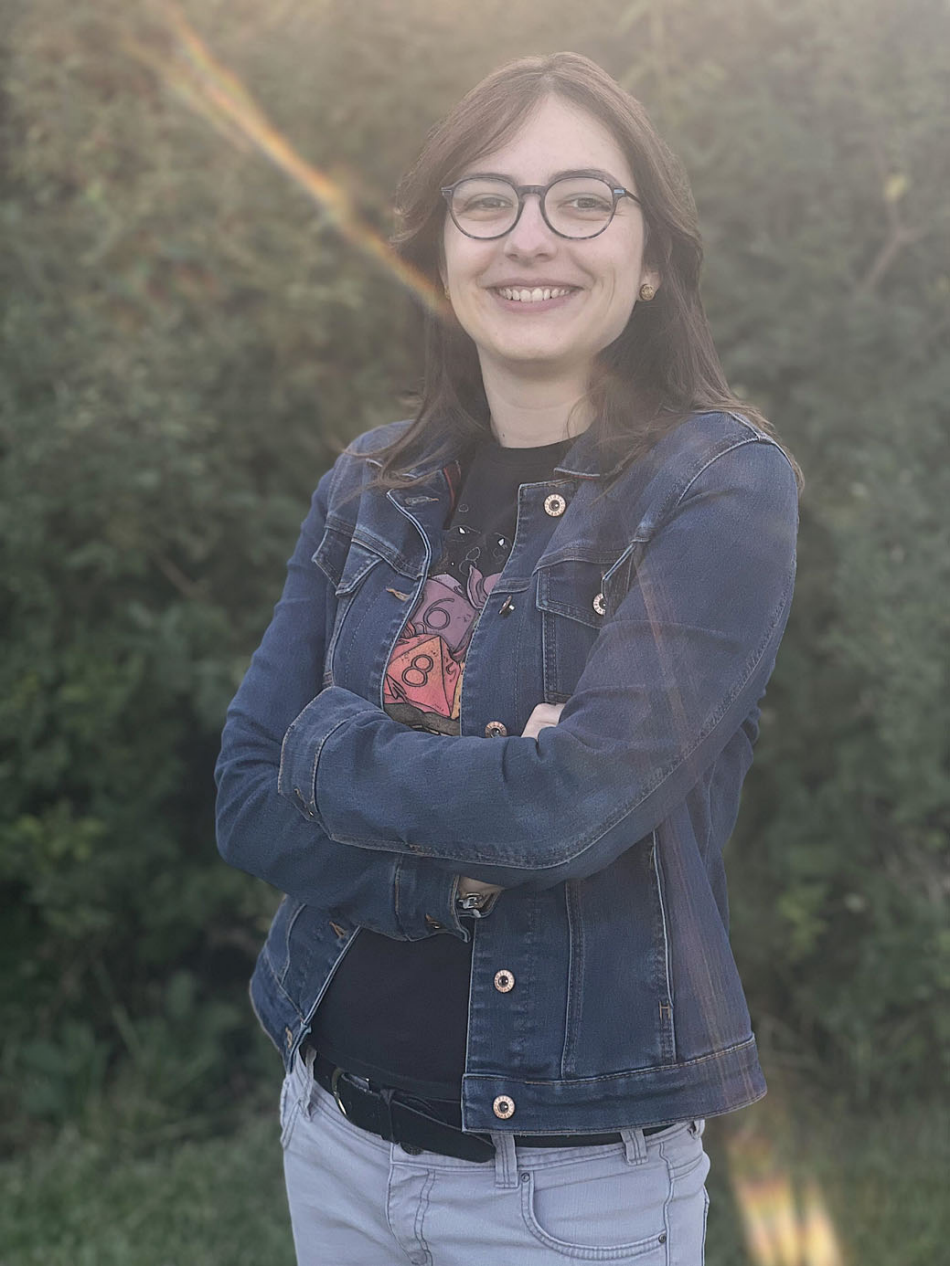


**Julie Frion**

**Describe your scientific journey and your current research focus**

I like to learn about all types of sciences except mathematics. For a while, I chose to keep my scientific profile versatile. My journey to molecular biology was forged based on my failures and my achievements in various fields. After high school, I studied to become a vet and… I failed miserably the entrance examination. But at the same time, I passed with flying colours the entrance examination for engineering school. So, I became an engineer in biotechnology with a specialization in therapeutic drug design. I tried several fields of research in molecular biology, mostly extremophiles bacteria and human cells. My journey is a fails-and-success story but I wouldn't trade it for anything.

I'm currently working on the characterization of a new protein that acts as a post-translational modification. The goal is to identify its functions in human cells.

My journey is a fails-and-success story but I wouldn't trade it for anything.

**Who or what inspired you to become a scientist?**

I must admit that I was a very curious child, always asking questions about everything. Thankfully, my parents cultivated my curiosity by buying me kid books explaining natural phenomena and animal/bug biology. I was also an avid viewer of the French TV show “C'est pas Sorcier !” (Translated as “It's not rocket science!”). This show was huge at the time and I'm pretty sure it had an impact for many current French scientists of my age. In the end, all those things greatly contributed to my choice of becoming a scientist!

**How would you explain the main finding of your paper?**

In the human body, there are tiny molecules capable of binding different components inside our cells. By doing so, they mark the component with a specific signal which decide the future fate of the component. One of those tiny molecules is called ubiquitin: it is known to be the building block for complex signals. For example, think of ubiquitin as a letter of the alphabet: alone, it can be a word (=signal) on its own. Put several letters together and you can create more complex words (=signals). Basically, in the human cells, several units of ubiquitin can form many signals: each signal will specifically trigger a different fate for the marked component. In previous work, we identified the equivalent of a new letter, the molecule Ub^KEKS^. Here, we investigated what type of signal can be formed by Ub^KEKS^ when it binds to components in a peculiar region of the human cell.

**What are the potential implications of this finding for your field of research?**

Our work is the first one to investigate the nucleolar functions of Ub^KEKS^. By identifying the functions of the variant, we bring a new layer of regulation in the already complex ubiquitin language. This provides a more complete view for better understanding cellular mechanisms which are still obscure. More, if a significant role is found in the regulation of a key pathway, Ub^KEKS^ could become a new target for specific treatment.

**Figure:**
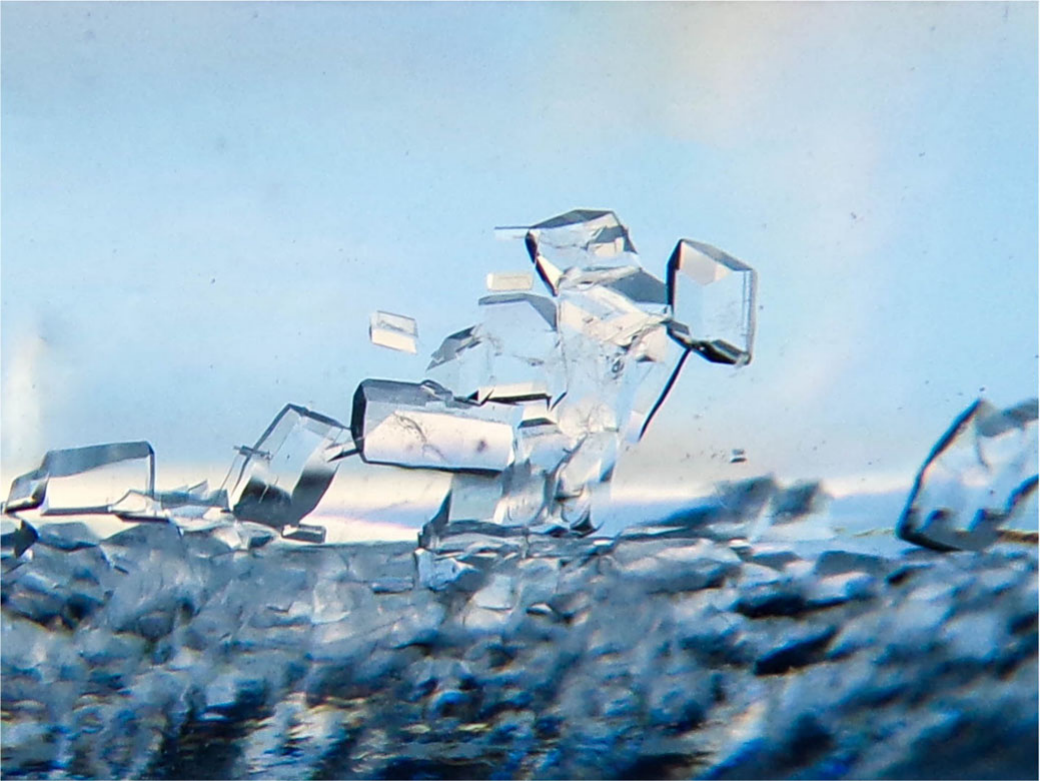
**Frosty ice skater.** This picture shows sucrose crystals formed in the solution used to isolate nucleoli of cancerous cells.

**Which part of this research project was the most rewarding?**

After several months of optimization, obtaining nucleolar extracts with a sufficient purity level for mass spectrometry was definitely a win!

**What do you enjoy most about being an early-career researcher?**

The best part is the possibility to switch your field of research whenever you change projects. It truly helps to discover which field may or may not suit your expectations; plus it gives a great first-hand overview of all the available tools.

**What piece of advice would you give to the next generation of researchers?**

I would give two pieces of advice. The first one is more for students: it is OK to try (and potentially fail) several fields before finding one that you like and where you are good at. As long as you're staying curious and motivated, you'll be able to lift mountains. The second one is more for researchers starting their career and above. Never underestimate the need for scientific communication, no matter the medium used to do so. It is so important, as it creates a vocation for the youth and improves the general public's trust in sciences. With the events of the last few years, I'm convinced that providing people with tools to develop their critical thinking is crucial.

I'm convinced that providing people with tools to develop their critical thinking is crucial.

**What's next for you?**

The next step is my thesis defence. After that, who knows where the road will take me.
